# Supplementary material for: Machine Learning Accelerates Crystallization for Structure Determination
Source: Angew Chem Int Ed Engl. 2026 May 4;65(25):e1218503. doi: 10.1002/anie.1218503 (PMC13266958; doi:10.1002/anie.1218503)

checkCIF/PLATON report

Structure factors have been supplied for datablock(s) lcz113

THIS REPORT IS FOR GUIDANCE ONLY. IF USED AS PART OF A REVIEW PROCEDURE FOR PUBLICATION, IT SHOULD NOT REPLACE THE EXPERTISE OF AN EXPERIENCED CRYSTALLOGRAPHIC REFEREE.

No syntax errors found. CIF dictionary Interpreting this report

Datablock: lcz113

Bond precision:	C-C = 0.0184 A		Wavelength=1.54184
Cell:	a=13.6323(4)	b=32.4016(9)	c=24.8156(6)
	alpha=90	beta=95.785(2)	gamma=90
Temperature:	100 K		

	Calculated	Reported
Volume	10905.4(5)	10905.4(5)
Space group	P 21	P 1 21 1
Hall group	P 2yb	P 2yb
Moiety formula	C141 H157 Ag9 F54 N18 O28 [+ solvent]	2(C15 H3 Ag3 F18 N6), C63 H77 Ag3 F18 N6 O14, C48 H74 O14
Sum formula	C141 H157 Ag9 F54 N18 O28 [+ solvent]	C141 H153 Ag9 F54 N18 O28
Mr	4548.64	4544.63
Dx,g cm-3	1.385	1.384
Z	2	2
Mu (mm-1)	7.246	7.246
F000	4524.0	4516.0
F000’	4543.47	
h,k,lmax	17,41,31	17,39,31
Nref	46740[23797]	36007
Tmin,Tmax	0.336,0.363	0.749,1.000
Tmin’	0.254	

Correction method= # Reported T Limits: Tmin=0.749 Tmax=1.000
AbsCorr = MULTI-SCAN

Data completeness= 1.51/0.77 Theta(max)= 78.137

R(reflections)= 0.0815(16688) wR2(reflections)= 0.2394(36007)

S = 0.940 Npar= 2196

The following ALERTS were generated. Each ALERT has the format
test-name_ALERT_alert-type_alert-level.
Click on the hyperlinks for more details of the test.

Alert level C

```
PLAT026_ALERT_3_C Ratio Observed / Unique Reflections (too) Low ..        46% Check
PLAT220_ALERT_2_C NonSolvent   Resd 1  C   Ueq(max)/Ueq(min) Range        3.3 Ratio
PLAT242_ALERT_2_C Low    'MainMol' Ueq as Compared to Neighbors of        Ag4 Check
PLAT242_ALERT_2_C Low    'MainMol' Ueq as Compared to Neighbors of        Ag6 Check
PLAT242_ALERT_2_C Low    'MainMol' Ueq as Compared to Neighbors of         O4 Check
PLAT260_ALERT_2_C Large Average Ueq of Residue Including       Ag1      0.118 Check
PLAT342_ALERT_3_C Low Bond Precision on  C-C Bonds ...............     0.0184 Ang.
PLAT360_ALERT_2_C Short  C(sp3)-C(sp3) Bond  C49      - C66      .       1.39 Ang.
PLAT360_ALERT_2_C Short  C(sp3)-C(sp3) Bond  C102     - C118     .       1.39 Ang.
PLAT410_ALERT_2_C Short Intra H...H Contact  H68      ..H80      .       1.99 Ang.
                                                      x,y,z  =      1_555 Check
PLAT410_ALERT_2_C Short Intra H...H Contact  H101     ..H113     .       1.99 Ang.
                                                      x,y,z  =      1_555 Check
PLAT413_ALERT_2_C Short Inter XH3 .. XHn     H10D     ..H63      .       2.09 Ang.
                                             2-x,1/2+y,2-z  =      2_757 Check
PLAT434_ALERT_2_C Short Inter HL..HL Contact F31      ..F49      .       2.53 Ang.
                                                    1+x,y,z  =      1_655 Check
PLAT733_ALERT_1_C Torsion Calc    16.5(13), Rep     16.5(3) ......       4.33 s.u.-R
             AG4 -N14 -C109-C137  1_555  1_555  1_555  1_555      #   18 Check
PLAT733_ALERT_1_C Torsion Calc   -179.9(8), Rep  179.95(19) ......       4.21 s.u.-R
             N3  -N4  -C9  -C50   1_555  1_555  1_555  1_555      #  133 Check
PLAT911_ALERT_3_C Missing FCF Refl Between Thmin & STh/L=    0.600         38 Report
               4 20  0,  -2  0  1,   0  2  1, -15  5  1,  -7  9  1,   0  0  2,
               1  0  2, -15  8  2,  13  9  2,  -5  0  3,   0  0  3, -15  9  3,
               6 21  4,  10  8  5,   7  6  6, -16  7  6,   7  5  7,  -3  0  8,
               4  4  9,   5  3 10,   2 22 10,   4  0 12,   3  0 13,  -8 28 16,
             -10  0 19, -10  0 20, -10  1 20,  10 11 20,   9 10 21,   9 11 21,
                 (    8 More Missing: see the .ckf listing file)
PLAT915_ALERT_3_C No Flack x Check Done: Low Friedel Pair Coverage         58 %
PLAT976_ALERT_2_C Check Calcd Resid. Dens.  0.77Ang From O7      .      -0.43 eA-3
```

Alert level G

```
FORMU01_ALERT_1_G  There is a discrepancy between the atom counts in the
            _chemical_formula_sum and _chemical_formula_moiety. This is
            usually due to the moiety formula being in the wrong format.
            Atom count from _chemical_formula_sum:   C141 H153 Ag9 F54 N18 O28
            Atom count from _chemical_formula_moiety:C141 H157 Ag9 F54 N18 O28
```

```
FORMU01_ALERT_2_G  There is a discrepancy between the atom counts in the
            _chemical_formula_sum and the formula from the _atom_site* data.
            Atom count from _chemical_formula_sum:C141 H153 Ag9 F54 N18 O28
            Atom count from the _atom_site data:  C141 H157 Ag9 F54 N18 O28
CELLZ01_ALERT_1_G Difference between formula and atom_site contents detected.
CELLZ01_ALERT_1_G ALERT: Large difference may be due to a
            symmetry error - see SYMMG tests
           From the CIF: _cell_formula_units_Z    2
           From the CIF: _chemical_formula_sum  C141 H153 Ag9 F54 N18 O28
           TEST: Compare cell contents of formula and atom_site data

           atom    Z*formula  cif sites diff
           C        282.00    282.00    0.00
           H        306.00    314.00   -8.00
           Ag        18.00     18.00    0.00
           F        108.00    108.00    0.00
           N         36.00     36.00    0.00
           O         56.00     56.00    0.00
PLAT002_ALERT_2_G Number of Distance or Angle Restraints on AtSite        251 Note
PLAT003_ALERT_2_G Number of Uiso or U(i,j)  Restrained non-H-Atoms        250 Report
PLAT007_ALERT_5_G Number of Unrefined Donor-H Atoms .............           6 Report
              H2    H4    H7    H13   H16   H22A
PLAT033_ALERT_4_G Flack x Value Deviates > 3.0 * Sigma from Zero .      0.162 Note
PLAT041_ALERT_1_G Calc. and Reported SumFormula    Strings  Differ     Please Check
              Calc: C141 H157 Ag9 F54 N18 O28
              Rep.: C141 H153 Ag9 F54 N18 O28
PLAT042_ALERT_1_G Calc. and Reported MoietyFormula Strings  Differ     Please Check
              Calc: C141 H157 Ag9 F54 N18 O28
              Rep.: 2(C15 H3 Ag3 F18 N6), C63 H77 Ag3 F18 N6 O14,
                    C48 H74 O14
PLAT072_ALERT_2_G SHELXL First  Parameter in WGHT  Unusually Large       0.12 Report
PLAT172_ALERT_4_G The CIF-Embedded .res File Contains DFIX Records        148 Report
PLAT173_ALERT_4_G The CIF-Embedded .res File Contains DANG Records        140 Report
PLAT174_ALERT_4_G The CIF-Embedded .res File Contains FLAT Records         10 Report
PLAT176_ALERT_4_G The CIF-Embedded .res File Contains SADI Records         74 Report
PLAT177_ALERT_4_G The CIF-Embedded .res File Contains DELU Records          5 Report
PLAT178_ALERT_4_G The CIF-Embedded .res File Contains SIMU Records          6 Report
PLAT186_ALERT_4_G The CIF-Embedded .res File Contains ISOR Records          5 Report
PLAT188_ALERT_3_G A Non-default SIMU Restraint Value has been used     0.0100 Report
PLAT188_ALERT_3_G A Non-default SIMU Restraint Value has been used     0.0100 Report
PLAT188_ALERT_3_G A Non-default SIMU Restraint Value has been used     0.0100 Report
PLAT188_ALERT_3_G A Non-default SIMU Restraint Value has been used     0.0100 Report
PLAT188_ALERT_3_G A Non-default SIMU Restraint Value has been used     0.0100 Report
PLAT191_ALERT_3_G A Non-default SADI Restraint Value has been used     0.0100 Report
PLAT191_ALERT_3_G A Non-default SADI Restraint Value has been used     0.0100 Report
PLAT191_ALERT_3_G A Non-default SADI Restraint Value has been used     0.0100 Report
PLAT191_ALERT_3_G A Non-default SADI Restraint Value has been used     0.0100 Report
PLAT191_ALERT_3_G A Non-default SADI Restraint Value has been used     0.0100 Report
PLAT191_ALERT_3_G A Non-default SADI Restraint Value has been used     0.0100 Report
PLAT191_ALERT_3_G A Non-default SADI Restraint Value has been used     0.0100 Report
PLAT191_ALERT_3_G A Non-default SADI Restraint Value has been used     0.0100 Report
PLAT191_ALERT_3_G A Non-default SADI Restraint Value has been used     0.0100 Report
PLAT191_ALERT_3_G A Non-default SADI Restraint Value has been used     0.0100 Report
PLAT191_ALERT_3_G A Non-default SADI Restraint Value has been used     0.0100 Report
PLAT191_ALERT_3_G A Non-default SADI Restraint Value has been used     0.0100 Report
PLAT191_ALERT_3_G A Non-default SADI Restraint Value has been used     0.0100 Report
PLAT191_ALERT_3_G A Non-default SADI Restraint Value has been used     0.0100 Report
```

```
PLAT191_ALERT_3_G A Non-default SADI Restraint Value has been used     0.0100 Report
PLAT191_ALERT_3_G A Non-default SADI Restraint Value has been used     0.0100 Report
PLAT191_ALERT_3_G A Non-default SADI Restraint Value has been used     0.0100 Report
PLAT191_ALERT_3_G A Non-default SADI Restraint Value has been used     0.0100 Report
PLAT191_ALERT_3_G A Non-default SADI Restraint Value has been used     0.0100 Report
PLAT191_ALERT_3_G A Non-default SADI Restraint Value has been used     0.0100 Report
PLAT191_ALERT_3_G A Non-default SADI Restraint Value has been used     0.0100 Report
PLAT191_ALERT_3_G A Non-default SADI Restraint Value has been used     0.0100 Report
PLAT191_ALERT_3_G A Non-default SADI Restraint Value has been used     0.0100 Report
PLAT191_ALERT_3_G A Non-default SADI Restraint Value has been used     0.0100 Report
PLAT191_ALERT_3_G A Non-default SADI Restraint Value has been used     0.0100 Report
PLAT191_ALERT_3_G A Non-default SADI Restraint Value has been used     0.0100 Report
PLAT191_ALERT_3_G A Non-default SADI Restraint Value has been used     0.0100 Report
PLAT191_ALERT_3_G A Non-default SADI Restraint Value has been used     0.0100 Report
PLAT191_ALERT_3_G A Non-default SADI Restraint Value has been used     0.0100 Report
PLAT191_ALERT_3_G A Non-default SADI Restraint Value has been used     0.0100 Report
PLAT191_ALERT_3_G A Non-default SADI Restraint Value has been used     0.0100 Report
PLAT191_ALERT_3_G A Non-default SADI Restraint Value has been used     0.0100 Report
PLAT191_ALERT_3_G A Non-default SADI Restraint Value has been used     0.0100 Report
PLAT191_ALERT_3_G A Non-default SADI Restraint Value has been used     0.0100 Report
PLAT191_ALERT_3_G A Non-default SADI Restraint Value has been used     0.0100 Report
PLAT191_ALERT_3_G A Non-default SADI Restraint Value has been used     0.0100 Report
PLAT191_ALERT_3_G A Non-default SADI Restraint Value has been used     0.0100 Report
PLAT191_ALERT_3_G A Non-default SADI Restraint Value has been used     0.0100 Report
PLAT191_ALERT_3_G A Non-default SADI Restraint Value has been used     0.0100 Report
PLAT191_ALERT_3_G A Non-default SADI Restraint Value has been used     0.0100 Report
PLAT191_ALERT_3_G A Non-default SADI Restraint Value has been used     0.0100 Report
PLAT191_ALERT_3_G A Non-default SADI Restraint Value has been used     0.0100 Report
PLAT191_ALERT_3_G A Non-default SADI Restraint Value has been used     0.0100 Report
PLAT191_ALERT_3_G A Non-default SADI Restraint Value has been used     0.0100 Report
PLAT191_ALERT_3_G A Non-default SADI Restraint Value has been used     0.0100 Report
PLAT191_ALERT_3_G A Non-default SADI Restraint Value has been used     0.0100 Report
PLAT191_ALERT_3_G A Non-default SADI Restraint Value has been used     0.0100 Report
PLAT191_ALERT_3_G A Non-default SADI Restraint Value has been used     0.0100 Report
PLAT191_ALERT_3_G A Non-default SADI Restraint Value has been used     0.0100 Report
PLAT191_ALERT_3_G A Non-default SADI Restraint Value has been used     0.0100 Report
PLAT191_ALERT_3_G A Non-default SADI Restraint Value has been used     0.0100 Report
PLAT191_ALERT_3_G A Non-default SADI Restraint Value has been used     0.0100 Report
PLAT191_ALERT_3_G A Non-default SADI Restraint Value has been used     0.0100 Report
PLAT191_ALERT_3_G A Non-default SADI Restraint Value has been used     0.0100 Report
PLAT191_ALERT_3_G A Non-default SADI Restraint Value has been used     0.0100 Report
PLAT191_ALERT_3_G A Non-default SADI Restraint Value has been used     0.0100 Report
PLAT191_ALERT_3_G A Non-default SADI Restraint Value has been used     0.0100 Report
PLAT191_ALERT_3_G A Non-default SADI Restraint Value has been used     0.0100 Report
PLAT191_ALERT_3_G A Non-default SADI Restraint Value has been used     0.0100 Report
PLAT191_ALERT_3_G A Non-default SADI Restraint Value has been used     0.0100 Report
PLAT191_ALERT_3_G A Non-default SADI Restraint Value has been used     0.0100 Report
PLAT191_ALERT_3_G A Non-default SADI Restraint Value has been used     0.0100 Report
PLAT191_ALERT_3_G A Non-default SADI Restraint Value has been used     0.0100 Report
PLAT191_ALERT_3_G A Non-default SADI Restraint Value has been used     0.0100 Report
PLAT191_ALERT_3_G A Non-default SADI Restraint Value has been used     0.0100 Report
PLAT191_ALERT_3_G A Non-default SADI Restraint Value has been used     0.0100 Report
PLAT191_ALERT_3_G A Non-default SADI Restraint Value has been used     0.0100 Report
PLAT191_ALERT_3_G A Non-default SADI Restraint Value has been used     0.0100 Report
PLAT191_ALERT_3_G A Non-default SADI Restraint Value has been used     0.0100 Report
PLAT191_ALERT_3_G A Non-default SADI Restraint Value has been used     0.0100 Report
PLAT191_ALERT_3_G A Non-default SADI Restraint Value has been used     0.0100 Report
```

```
PLAT191_ALERT_3_G A Non-default SADI Restraint Value has been used     0.0100 Report
PLAT191_ALERT_3_G A Non-default SADI Restraint Value has been used     0.0100 Report
PLAT191_ALERT_3_G A Non-default SADI Restraint Value has been used     0.0100 Report
PLAT192_ALERT_3_G A Non-default DELU Restraint Value for SecondPar     0.0200 Report
PLAT192_ALERT_3_G A Non-default DELU Restraint Value for SecondPar     0.0200 Report
PLAT192_ALERT_3_G A Non-default DELU Restraint Value for SecondPar     0.0200 Report
PLAT192_ALERT_3_G A Non-default DELU Restraint Value for SecondPar     0.0200 Report
PLAT192_ALERT_3_G A Non-default DELU Restraint Value for SecondPar     0.0200 Report
PLAT242_ALERT_2_G Low    'MainMol' Ueq as Compared to Neighbors of        C13 Check
PLAT242_ALERT_2_G Low    'MainMol' Ueq as Compared to Neighbors of        C29 Check
PLAT242_ALERT_2_G Low    'MainMol' Ueq as Compared to Neighbors of        C33 Check
PLAT242_ALERT_2_G Low    'MainMol' Ueq as Compared to Neighbors of        C42 Check
PLAT242_ALERT_2_G Low    'MainMol' Ueq as Compared to Neighbors of        C48 Check
PLAT242_ALERT_2_G Low    'MainMol' Ueq as Compared to Neighbors of        C50 Check
PLAT242_ALERT_2_G Low    'MainMol' Ueq as Compared to Neighbors of        C91 Check
PLAT242_ALERT_2_G Low    'MainMol' Ueq as Compared to Neighbors of       C103 Check
PLAT242_ALERT_2_G Low    'MainMol' Ueq as Compared to Neighbors of       C129 Check
PLAT242_ALERT_2_G Low    'MainMol' Ueq as Compared to Neighbors of       C130 Check
PLAT242_ALERT_2_G Low    'MainMol' Ueq as Compared to Neighbors of       C131 Check
PLAT242_ALERT_2_G Low    'MainMol' Ueq as Compared to Neighbors of       C132 Check
PLAT242_ALERT_2_G Low    'MainMol' Ueq as Compared to Neighbors of       C133 Check
PLAT242_ALERT_2_G Low    'MainMol' Ueq as Compared to Neighbors of       C134 Check
PLAT242_ALERT_2_G Low    'MainMol' Ueq as Compared to Neighbors of       C135 Check
PLAT242_ALERT_2_G Low    'MainMol' Ueq as Compared to Neighbors of       C136 Check
PLAT242_ALERT_2_G Low    'MainMol' Ueq as Compared to Neighbors of       C137 Check
PLAT242_ALERT_2_G Low    'MainMol' Ueq as Compared to Neighbors of       C138 Check
PLAT398_ALERT_2_G Deviating  C-O-C   Angle From 120 for O18      .      108.8 Degree
PLAT398_ALERT_2_G Deviating  C-O-C   Angle From 120 for O28      .      109.7 Degree
PLAT606_ALERT_4_G Solvent Accessible VOID(S) in  Crystal Structure          ! Info
PLAT791_ALERT_4_G Model has Chirality at C4         (Sohncke SpGr)          S Verify
PLAT791_ALERT_4_G Model has Chirality at C5         (Sohncke SpGr)          R Verify
PLAT791_ALERT_4_G Model has Chirality at C12        (Sohncke SpGr)          R Verify
PLAT791_ALERT_4_G Model has Chirality at C27        (Sohncke SpGr)          R Verify
PLAT791_ALERT_4_G Model has Chirality at C30        (Sohncke SpGr)          R Verify
PLAT791_ALERT_4_G Model has Chirality at C31        (Sohncke SpGr)          R Verify
PLAT791_ALERT_4_G Model has Chirality at C32        (Sohncke SpGr)          S Verify
PLAT791_ALERT_4_G Model has Chirality at C39        (Sohncke SpGr)          R Verify
PLAT791_ALERT_4_G Model has Chirality at C40        (Sohncke SpGr)          S Verify
PLAT791_ALERT_4_G Model has Chirality at C44        (Sohncke SpGr)          R Verify
PLAT791_ALERT_4_G Model has Chirality at C45        (Sohncke SpGr)          R Verify
PLAT791_ALERT_4_G Model has Chirality at C47        (Sohncke SpGr)          S Verify
PLAT791_ALERT_4_G Model has Chirality at C51        (Sohncke SpGr)          R Verify
PLAT791_ALERT_4_G Model has Chirality at C52        (Sohncke SpGr)          S Verify
PLAT791_ALERT_4_G Model has Chirality at C56        (Sohncke SpGr)          S Verify
PLAT791_ALERT_4_G Model has Chirality at C57        (Sohncke SpGr)          R Verify
PLAT791_ALERT_4_G Model has Chirality at C60        (Sohncke SpGr)          S Verify
PLAT791_ALERT_4_G Model has Chirality at C65        (Sohncke SpGr)          S Verify
PLAT791_ALERT_4_G Model has Chirality at C67        (Sohncke SpGr)          S Verify
PLAT791_ALERT_4_G Model has Chirality at C68        (Sohncke SpGr)          S Verify
PLAT791_ALERT_4_G Model has Chirality at C71        (Sohncke SpGr)          R Verify
PLAT791_ALERT_4_G Model has Chirality at C72        (Sohncke SpGr)          S Verify
PLAT791_ALERT_4_G Model has Chirality at C77        (Sohncke SpGr)          S Verify
PLAT791_ALERT_4_G Model has Chirality at C78        (Sohncke SpGr)          S Verify
PLAT791_ALERT_4_G Model has Chirality at C80        (Sohncke SpGr)          R Verify
PLAT791_ALERT_4_G Model has Chirality at C84        (Sohncke SpGr)          R Verify
PLAT791_ALERT_4_G Model has Chirality at C85        (Sohncke SpGr)          S Verify
PLAT791_ALERT_4_G Model has Chirality at C86        (Sohncke SpGr)          S Verify
```

```
PLAT791_ALERT_4_G Model has Chirality at C89       (Sohncke SpGr)          S Verify
PLAT791_ALERT_4_G Model has Chirality at C92       (Sohncke SpGr)          S Verify
PLAT791_ALERT_4_G Model has Chirality at C93       (Sohncke SpGr)          S Verify
PLAT791_ALERT_4_G Model has Chirality at C95       (Sohncke SpGr)          S Verify
PLAT791_ALERT_4_G Model has Chirality at C101      (Sohncke SpGr)          R Verify
PLAT791_ALERT_4_G Model has Chirality at C112      (Sohncke SpGr)          S Verify
PLAT791_ALERT_4_G Model has Chirality at C113      (Sohncke SpGr)          S Verify
PLAT791_ALERT_4_G Model has Chirality at C116      (Sohncke SpGr)          R Verify
PLAT791_ALERT_4_G Model has Chirality at C117      (Sohncke SpGr)          S Verify
PLAT791_ALERT_4_G Model has Chirality at C119      (Sohncke SpGr)          S Verify
PLAT791_ALERT_4_G Model has Chirality at C121      (Sohncke SpGr)          S Verify
PLAT791_ALERT_4_G Model has Chirality at C139      (Sohncke SpGr)          R Verify
PLAT860_ALERT_3_G Number of Least-Squares Restraints ............       4414 Note
PLAT868_ALERT_4_G ALERTS Due to the Use of _smtbx_masks Suppressed          ! Info
PLAT883_ALERT_1_G Absent Datum for _atom_sites_solution_primary ..     Please Do !
PLAT910_ALERT_3_G Missing FCF Reflection(s) Below Theta(Min)[Deg]=       2.73 Note
                0  0  1,   0  1  1,
PLAT912_ALERT_4_G Missing # of FCF Reflections Above STh/L=  0.600        945 Note
PLAT916_ALERT_2_G Hooft y and Flack x Parameter Values Differ by .       0.13 Check
PLAT933_ALERT_2_G Number of HKL-OMIT Records in Embedded .res File        101 Note
               -6 12 10,  -6 14 12,  -5  0  3,  -3  0  8,   0 -2  1,   0  0  2,
                0  0  3,   0  2  1,   2-15  6,   2-12 23,   3-14 23,   3-13 22,
                3-13 23,   3-12 22,   3-12 23,   3-12 24,   3-11 23,   3-11 24,
                4-15 22,   4-14 22,   4-14 23,   4-13 22,   4-13 23,   4-13 24,
                4-12 23,   4-12 24,   4-11 19,   4-11 20,   4-11 24,   4-10 25,
                5-15 21,   5-15 22,   5-14 22,   5-14 23,   5-13 23,   5-13 24,
                5-12 20,   5-12 23,   5-12 24,   5-11 20,   5-11 24,   5-10 20,
                5-10 21,   6-15 21,   6-14 19,   6-14 22,   6-14 23,   6-13 19,
                6-13 20,   6-13 23,
PLAT941_ALERT_3_G Average HKL Measurement Multiplicity ..........         3.0 Low
PLAT951_ALERT_5_G Calculated (ThMax) and CIF-Reported Kmax Differ           2 Units
PLAT957_ALERT_1_G Calculated (ThMax) and Actual (FCF) Kmax Differ           2 Units
PLAT969_ALERT_5_G The 'Henn et al.' R-Factor-gap value ..........      2.430 Note
              Predicted wR2: Based on SigI**2  9.85 or SHELX Weight 25.46
PLAT978_ALERT_2_G Number C-C Bonds with Positive Residual Density.          0 Info
```

```
   0 ALERT level A = Most likely a serious problem - resolve or explain
   0 ALERT level B = A potentially serious problem, consider carefully
  18 ALERT level C = Check. Ensure it is not caused by an omission or oversight
 175 ALERT level G = General information/check it is not something unexpected

   9 ALERT type 1 CIF construction/syntax error, inconsistent or missing data
  39 ALERT type 2 Indicator that the structure model may be wrong or deficient
  91 ALERT type 3 Indicator that the structure quality may be low
  51 ALERT type 4 Improvement, methodology, query or suggestion
   3 ALERT type 5 Informative message, check
```

Validation response form

Please find below a validation response form (VRF) that can be filled in and pasted into your CIF.

```
# start Validation Reply Form
_vrf_PLAT026_lcz113
;
PROBLEM: Ratio Observed / Unique Reflections (too) Low ..        46% Check
```

```
RESPONSE: ...
;
_vrf_PLAT220_lcz113
;
PROBLEM: NonSolvent   Resd 1  C   Ueq(max)/Ueq(min) Range        3.3 Ratio
RESPONSE: ...
;
_vrf_PLAT242_lcz113
;
PROBLEM: Low    'MainMol' Ueq as Compared to Neighbors of        Ag4 Check
RESPONSE: ...
;
_vrf_PLAT260_lcz113
;
PROBLEM: Large Average Ueq of Residue Including       Ag1      0.118 Check
RESPONSE: ...
;
_vrf_PLAT342_lcz113
;
PROBLEM: Low Bond Precision on  C-C Bonds ...............     0.0184 Ang.
RESPONSE: ...
;
_vrf_PLAT360_lcz113
;
PROBLEM: Short  C(sp3)-C(sp3) Bond  C49      - C66      .       1.39 Ang.
RESPONSE: ...
;
_vrf_PLAT410_lcz113
;
PROBLEM: Short Intra H...H Contact  H68      ..H80      .       1.99 Ang.
RESPONSE: ...
;
_vrf_PLAT413_lcz113
;
PROBLEM: Short Inter XH3 .. XHn     H10D     ..H63      .       2.09 Ang.
RESPONSE: ...
;
_vrf_PLAT434_lcz113
;
PROBLEM: Short Inter HL..HL Contact F31      ..F49      .       2.53 Ang.
RESPONSE: ...
;
_vrf_PLAT733_lcz113
;
PROBLEM: Torsion Calc    16.5(13), Rep     16.5(3) ......       4.33 s.u.-R
RESPONSE: ...
;
_vrf_PLAT911_lcz113
;
PROBLEM: Missing FCF Refl Between Thmin & STh/L=    0.600         38 Report
RESPONSE: ...
;
_vrf_PLAT915_lcz113
;
PROBLEM: No Flack x Check Done: Low Friedel Pair Coverage         58 %
RESPONSE: ...
;
```

```
_vrf_PLAT976_lcz113
;
PROBLEM: Check Calcd Resid. Dens.  0.77Ang From O7      .      -0.43 eA-3
RESPONSE: ...
;
# end Validation Reply Form
```

It is advisable to attempt to resolve as many as possible of the alerts in all categories. Often the minor alerts point to easily fixed oversights, errors and omissions in your CIF or refinement strategy, so attention to these fine details can be worthwhile. It is up to the individual to critically assess their own results and, if necessary, seek expert advice.

PLATON version of 17/09/2025; check.def file version of 17/09/2025

---

duplicate check

No duplication found

Datablock lcz113 - ellipsoid plot

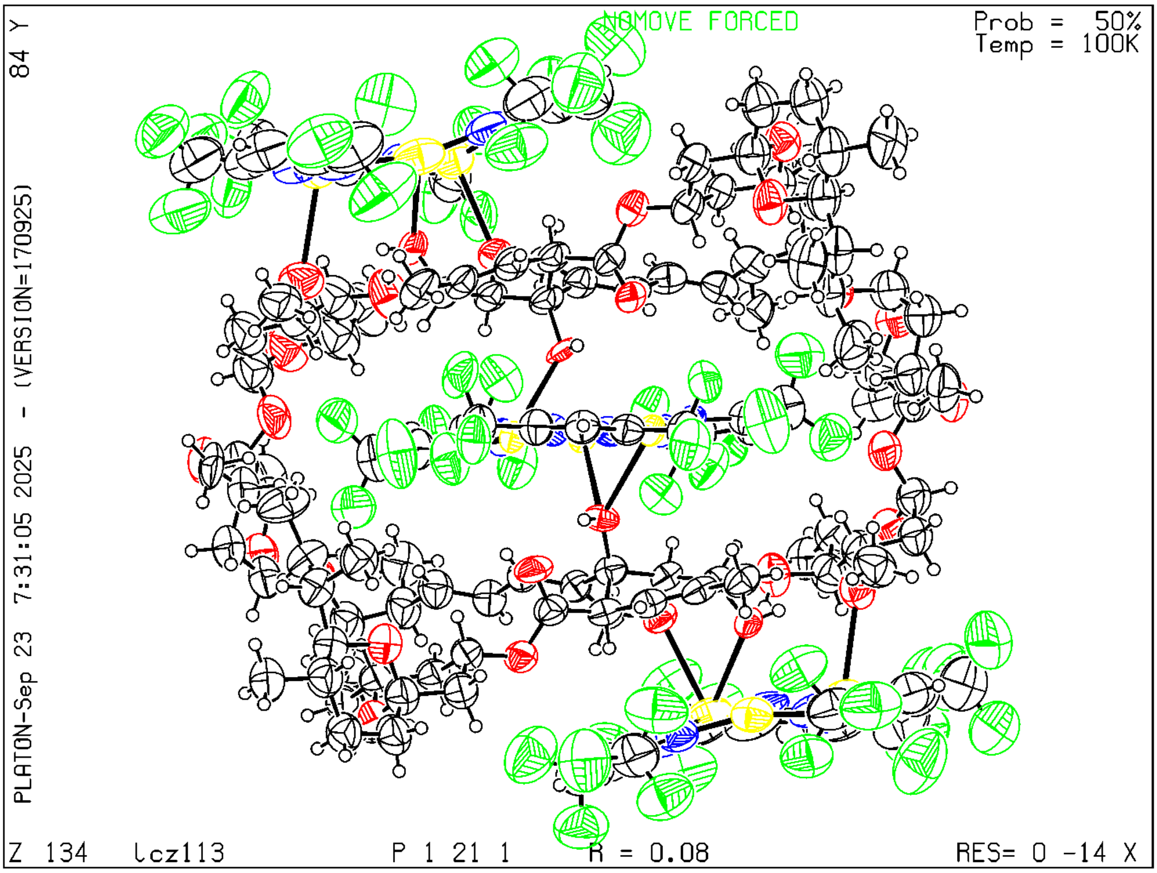

Supplement: Supplementary file 2 — Supporting File 2: anie72462‐sup‐0002‐DataFile.zip. [file ANIE-65-e1218503-s002.zip › supplementary_materials_crystallographic_data submit.zip!/cif_and_checkcif(1-114)/Ag3Pz3_113/Ag3Pz3_113_checkcif.pdf]
